# Supplementary material for: Hybrid Models and Biological Model Reduction with PyDSTool
Source: PLoS Comput Biol. 2012 Aug 9;8(8):e1002628. doi: 10.1371/journal.pcbi.1002628 (PMC3415397; doi:10.1371/journal.pcbi.1002628)
Supplement: Text S4 — Complete source code for the PyDSTool package (version 0.88.120504). Includes API documentation and help files linking to web pages. This file is identical to the current public release on Sourceforge.net. (ZIP) [file pcbi.1002628.s004.zip › PyDSTool/html/PyDSTool.Toolbox.event_driven_simulator.map1D-class.html]

xml version="1.0" encoding="ascii"?


PyDSTool.Toolbox.event\_driven\_simulator.map1D


| Home | Trees | Indices | Help | PyDSTool |
| --- | --- | --- | --- | --- |

Package PyDSTool ::
Package Toolbox ::
Module event\_driven\_simulator ::
Class map1D

[hide private]

[frames] | no frames]

# Class map1D

source code

```
object --+    
         |    
       map --+
             |
            map1D
```

Known Subclasses:
:   - composed\_map1D
    - , delay\_map
    - , identity\_map

---


|  |  |
| --- | --- |
| Instance Methods | [hide private] |

**Inherited from `object`**:
`__delattr__`,
`__getattribute__`,
`__hash__`,
`__init__`,
`__new__`,
`__reduce__`,
`__reduce_ex__`,
`__repr__`,
`__setattr__`,
`__str__`


|  |  |
| --- | --- |
| Properties | [hide private] |

**Inherited from `object`**:
`__class__`

| Home | Trees | Indices | Help | PyDSTool |
| --- | --- | --- | --- | --- |

|  |  |
| --- | --- |
| Generated by Epydoc 3.0.1 on Fri May 4 15:24:08 2012 | http://epydoc.sourceforge.net |
